# Blood DNA methylation and breast cancer risk: a meta-analysis of four prospective cohort studies

**DOI:** 10.1186/s13058-019-1145-9

**Published:** 2019-05-17

**Authors:** Clara Bodelon, Srikant Ambatipudi, Pierre-Antoine Dugué, Annelie Johansson, Joshua N. Sampson, Belynda Hicks, Eric Karlins, Amy Hutchinson, Cyrille Cuenin, Veronique Chajès, Melissa C. Southey, Isabelle Romieu, Graham G. Giles, Dallas English, Silvia Polidoro, Manuela Assumma, Laura Baglietto, Paolo Vineis, Gianluca Severi, Zdenko Herceg, James M. Flanagan, Roger L. Milne, Montserrat Garcia-Closas

**Affiliations:** 1 Divison of Cancer Epidemiology and Genetics, National Cancer Institute, Bethesda, USA; 2 International Agency for Research on Cancer (IARC), Lyon, France; 3 AMCHSS, Sree Chitra Tirunal Institute for Medical Sciences and Technology, Trivandrum, India; 4 Cancer Epidemiology and Intelligence Division, Cancer Council Victoria, Melbourne, Victoria Australia; 5 Centre for Epidemiology and Biostatistics, Melbourne School of Population and Global Health, The University of Melbourne, Melbourne, Victoria Australia; 6 Division of Cancer, Imperial College London, London, UK; 7 Cancer Genomics Research Laboratory, Leidos Biomedical Research, Inc., Frederick National Laboratory for Cancer Research, Frederick, USA; 8 Genetic Epidemiology Laboratory, Department of Pathology, The University of Melbourne, Parkville, Australia; 9 IIGM (Italian Institute for Genomic Medicine), Turin, Italy; 10 Department of Medical Sciences, University of Turin, Turin, Italy; 11 Department of Clinical and Experimental Medicine, University of Pisa, Pisa, Italy; 12 MRC-PHE Center for Environment and Health, School of Public Health, Imperial College, London, UK; 13 CESP (U1018 INSERM, Équipe Générations et Santé), Facultés de médecine Université Paris-Sud, UVSQ, Université Paris-Saclay, Villejuif, France

**Keywords:** Breast cancer risk, Blood DNA methylation, Prospective study, Meta-analysis

## Abstract

**Background:**

Environmental and genetic factors play an important role in the etiology of breast cancer. Several small blood-based DNA methylation studies have reported risk associations with methylation at individual CpGs and average methylation levels; however, these findings require validation in larger prospective cohort studies. To investigate the role of blood DNA methylation on breast cancer risk, we conducted a meta-analysis of four prospective cohort studies, including a total of 1663 incident cases and 1885 controls, the largest study of blood DNA methylation and breast cancer risk to date.

**Methods:**

We assessed associations with methylation at 365,145 CpGs present in the HumanMethylation450 (HM450K) Beadchip, after excluding CpGs that did not pass quality controls in all studies. Each of the four cohorts estimated odds ratios (ORs) and 95% confidence intervals (CI) for the association between each individual CpG and breast cancer risk. In addition, each study assessed the association between average methylation measures and breast cancer risk, adjusted and unadjusted for cell-type composition. Study-specific ORs were combined using fixed-effect meta-analysis with inverse variance weights. Stratified analyses were conducted by age at diagnosis (< 50, ≥ 50), estrogen receptor (ER) status (+/−), and time since blood collection (< 5, 5–10, > 10 years). The false discovery rate (*q* value) was used to account for multiple testing.

**Results:**

The average age at blood draw ranged from 52.2 to 62.2 years across the four cohorts. Median follow-up time ranged from 6.6 to 8.4 years. The methylation measured at individual CpGs was not associated with breast cancer risk (*q* value > 0.59). In addition, higher average methylation level was not associated with risk of breast cancer (OR = 0.94, 95% CI = 0.85, 1.05; *P* = 0.26; *P* for study heterogeneity = 0.86). We found no evidence of modification of this association by age at diagnosis (*P* = 0.17), ER status (*P* = 0.88), time since blood collection (*P* = 0.98), or CpG location (*P* = 0.98).

**Conclusions:**

Our data indicate that DNA methylation measured in the blood prior to breast cancer diagnosis in predominantly postmenopausal women is unlikely to be associated with substantial breast cancer risk on the HM450K array. Larger studies or with greater methylation coverage are needed to determine if associations exist between blood DNA methylation and breast cancer risk.

**Electronic supplementary material:**

The online version of this article (10.1186/s13058-019-1145-9) contains supplementary material, which is available to authorized users.

## Introduction

There are many well-established environmental and genetic factors associated with breast cancer risk but whether DNA methylation measured in blood is associated with breast cancer risk remains to be determined. The rationale for such an association is that blood DNA methylation levels could be a surrogate for breast tissue methylation [1–4], represent immunity/inflammation at the target tissue itself, indicate altered molecular pathways involved in carcinogenesis, or reflect past endogenous or exogenous exposures such as hormone levels [5, 6], alcohol [7], body mass index (BMI) [8, 9], smoking [10, 11], or ionizing radiation [12]. To better understand the possible relationship between blood DNA methylation and breast cancer risk, large, prospective studies with good exposure information and blood samples collected prior to diagnosis are needed.

Evidence for an association between blood-based DNA methylation and breast cancer risk is inconsistent [13]. A recent systematic review summarized the most promising findings of DNA methylation as a biomarker of disease [14], concluding that hypermethylation at *BRCA1* and *RASSF1A* gene promoters, and hypermethylation of the body of the *ATM* gene were more common in breast cancer cases [1–4, 15–21]. An analysis published after the systematic review reported CpG sites cg06418238 in *RPTOR*, cg00736299 in *MGRN1*, and cg27466532 in *RAPSN* to be more commonly hypomethylated in breast cancer cases [22], although these associations were not replicated by the Melbourne Collaborative Cohort Study (MCCS) [23–25]. A nested case-control study (298 cases and 612 controls) within the Sister Study [26] reported that methylation level at 250 CpG sites of the 27 K methylation array was associated with breast cancer. Global methylation refers to the total level of 5-methylcytosine (5mC) content in a sample relative to total cytosine [27] and global hypomethylation has been related to genomic instability and cancer development [9, 28]. This global measure has also been investigated as a potential biomarker for breast cancer risk, producing inconsistent findings [14, 29]. This could be partly explained by the use of different technologies and proxies to measure global methylation [27]. Most recently, methylation arrays have been used to measure selected methylation sites across the genome, and some studies have analyzed the average methylation across these sites as a surrogate measure of global methylation. Using this measure of global methylation derived for the HumanMethylation450 BeadChip array, two prospective studies, the MCCS [23] and the Italian cohort of the European Prospective Investigation into Cancer and Nutrition (EPIC-Italy) [30], reported that lower global methylation levels in blood DNA were associated with a higher risk of breast cancer. However, this association was not observed by the Norwegian Women and Cancer (NOWAC) study [30].

In this study, we examined the association between DNA methylation in blood and breast cancer risk in the largest to-date study, involving 1663 incident invasive breast cancer cases and 1885 controls combined from four prospective cohort studies.

## Methods

We combined results from four prospective studies: the MCCS [23], EPIC-Italy [30], the IARC cohort of the European Prospective Investigation into Cancer and Nutrition (EPIC-IARC) [31], and the Prostate, Lung, Colorectal, and Ovarian Cancer Screening Trial (PLCO) [32]. Details of the four cohorts and the case-control studies nested therein can be found in Additional file 1: Supplemental Note.

### Methylation array

Three of the cohorts (MCCS, EPIC-Italy, EPIC-IARC) measured DNA methylation using the Illumina Infinium HumanMethylation450 BeadChip (HM450K) array while PLCO used the MethylationEPIC BeadChip, which includes 90% of the sites in the HM450K array [33]. The four studies processed methylation data using the same protocol. Raw intensity data were imported into the R programming software using the *minfi* Bioconductor package [34]. Data were pre-processed for background subtraction and control normalization using the *preprocessIllumina* function. Subset-quantile within array normalization (SWAN) was performed to correct for type I and II probe bias [35]. Each study excluded samples for which more than 5% of CpG sites had a detection *P* value > 0.01. Also, each study excluded CpGs with a *P* value > 0.01 in more than 20% of the samples, and CpGs labeled as cross-reactive [36, 37], or located on the Y chromosome. The meta-analysis was performed on the 365,145 included CpGs common to the four studies. Beta values were used for analysis.

### Statistical analysis

The four studies individually performed conditional (MCCS, EPIC-Italy, EPIC-IARC) or unconditional (PLCO) logistic regression to estimate the odds ratio (OR) and associated standard error (SE) per one standard deviation increase in methylation for each of the 365,145 CpG probes, adjusting where appropriate for the matching variables specific to each study (see Additional file 1: Supplemental Note), cell-type proportions estimated with the Houseman algorithm (percentage CD8T+, CD4T+, NK, B cell, monocytes, granulocytes) [38], and other variables to account for batch effects, such as plate or surrogate variable analysis (SVA) [39, 40]. These analyses within each study were repeated for subsets of individuals defined by the following case characteristics: age at diagnosis (< 50, ≥ 50 years old), estrogen receptor (ER) status (ER+/−), stage (early and late, see Additional file 1: Supplemental Note; associations with individual CpG probes were done in the PLCO only) and time since from blood collection (< 2 years, ≥ 2 years; < 5, 5–10, > 10 years). Estimates of the pooled ORs and their associated SE were calculated using fixed-effects meta-analysis. The Wald test was used to calculate *P* values. Statistical significance was defined by a false discovery rate (FDR)-adjusted *P* value, or *q* value, less than 0.05, which means that 5% of significant tests (i.e., tests with *q* < 0.05) were expected to be false positives. Heterogeneity in the odds ratio across studies or subgroups was assessed using the *I*^2^ statistic and the *P* value for the Cochran’s *Q* statistic.

To assess enrichment at specific genomic regions, we considered the average of the ORs for the CpGs located in those regions. We defined the average log (OR), *β*_avg_, by *β*_avg_ *=* $\frac{1}{\mathrm{K}}\sum {\beta}_k$ and approximated its standard error, SE_avg_, by SE_avg_ *=* $\frac{1}{K^2}$ *w*^*T*^*ΦθΦw*, where *K* is the number of CpGs in the region of interest, *β*_*k*_ and *σ*_*k*_ the estimated log (OR), and SE for probe *k*, *θ* the *K* × *K* correlation matrix of the *K* probes among controls, *Φ* the *K* × *K* diagonal matrix with the SEs along the diagonal and *w* a column vector of values equal to 1. We then report exp(*β*_avg_) as the average OR and calculate the 95% confidence interval under normality assumption and *P* values using the Wald test.

Each of the four studies also performed adjusted logistic regression to assess the association between a one standard deviation increase in global DNA methylation and the risk of breast cancer. Global methylation was defined for each study participant as the average methylation level across all 365,145 CpGs. In addition, we computed average measures in CpG islands, CpG shores, CpG shelves, and “open sea,” as well as at regulatory regions defined using the UCSC classification (promoter, gene body, 3′ UTR or intergenic) [41]. CpG sites were considered to be in a promoter region if they were located within 1500 bp of the transcription start site (TSS), in the 5′UTR or in the first exon [41].

All analyses were conducted using the R software, version 3.4.1.

## Results

The characteristics of the 1663 cases and 1885 controls included in the meta-analysis are described in Table 1.

**Table 1:** Characteristics of the nested case-control studies included in the meta-analysis

Characteristics	Melbourne Collaborative Cohort Study	European Prospective Investigation into Cancer and Nutrition (Italy)	European Prospective Investigation into Cancer and Nutrition (IARC)	Prostate, Lung, Colorectal and Ovarian Screening Trial
Acronym	MCCS	EPIC-Italy	EPIC-IARC	PLCO
Reference	(17)	(18)	(19)	–
Location	Australia	Italy	Germany, Greece, Italy, Spain, The Netherlands, and the UK	US
Methylation array used	HM450K	HM450K	HM450K	EPIC
Number of subjects, *n*				
Controls (*N* = 1926)	409	248	423	805
Cases (*N* = 1703)	409	248	423	583
Age at blood draw (years), mean (SD)	56.7 (7.9)	52.2 (7.2)	52.2 (9.0)	62.2 (5.2)
Time from blood draw to diagnosis in cases				
Median (IQR)	7.7 (4.4., 11.1)	6.55 (2.5, 10.6)	7.7 (5.0, 10.3)	8.4 (5.6, 10.5)
Average (SD)	7.6 (3.9)	6.7 (4.4)	7.5 (3.2)	7.9 (3.5)
ER status, *n* (%)				
Positive	297 (72.6)	147 (59.3)	350 (82.7)	411 (70.5)
Negative	103 (25.2)	30 (12.0)	73 (17.3)	78 (12.9)
Stage^†^, *n* (%)				
Early	246 (60.1)	71 (28.6)	207 (48.9)	337 (57.8)
Late	141 (34.5)	40 (16.1)	95 (22.5)	183 (31.4)

*SD* standard deviation, *IQR* interquartile range, *ER* estrogen receptor

^†^Stage: a cancer was considered an early stage if it was classified as localized (EPIC-Italy, EPIC-IARC) or stage I (MCCS, PLCO). A cancer was considered late-stage if it was classified as regional or metastatic (EPIC-Italy, EPIC-IARC) or stages II, III, or IV (MCCS, PLCO)

### Associations between individual CpG sites and breast cancer risk

**Fig. 1:**
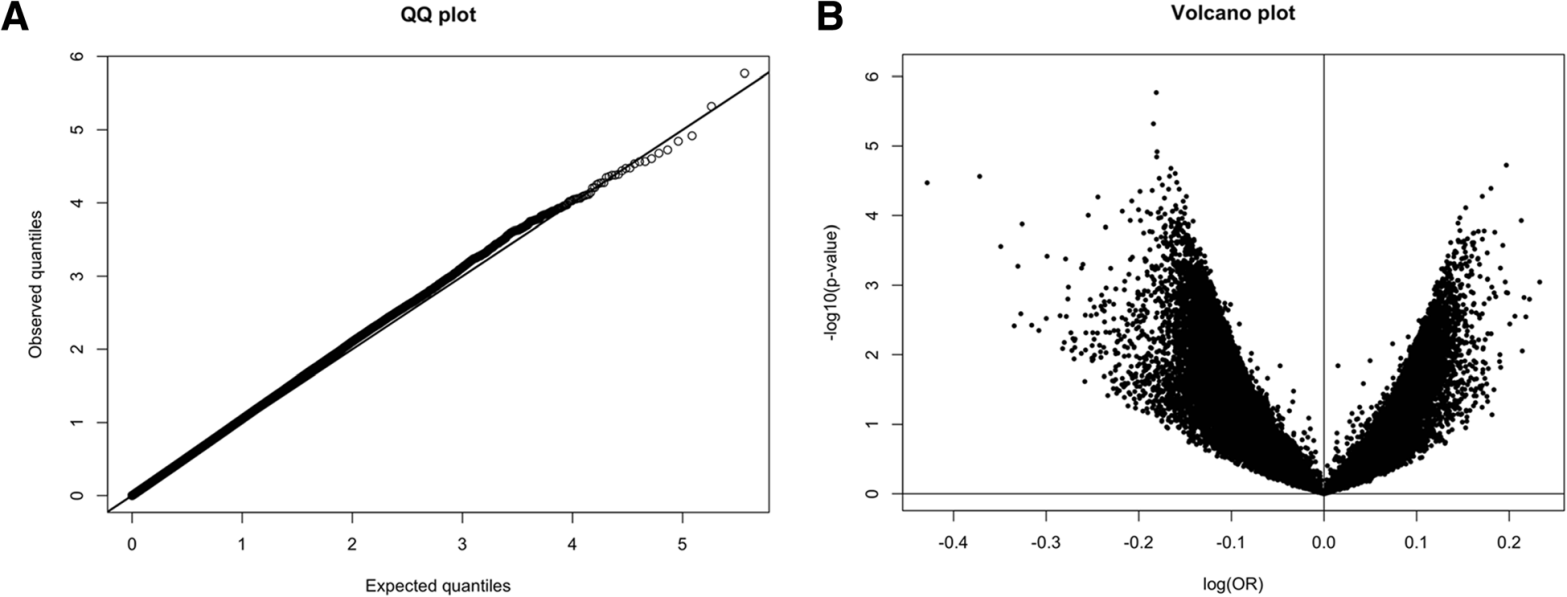
Overall associations between methylation and breast cancer risk. QQ-plot (**a**) and a volcano plot (**b**) for the overall associations between methylation values at individual CpG sites and breast cancer risk

We found no evidence of association between blood DNA methylation measured at individual CpG sites and breast cancer risk (Fig. 1, *q* > 0.59) overall, or after stratification by age (< 50, > 50 years old), ER status (ER+/−), stage (early/late), or time since blood collection (< 5, 5–10, > 10 years) (data not shown). To account for possible subclinical effects from occult disease at the time of blood collection, we also investigated risk associations < 2 years versus ≥ 2 years since blood draw. We observed putative associations within 2 years of blood draw with cg00899463 (promoter of *SGMS2*, *q* < 10^−8^) and cg07145930 (promoter of *G3BP1*, *q* = 5.78 × 10^−5^), but there was substantial heterogeneity across studies for both CpGs (*I*^2^ = 100%), with each association driven by a single study.

We next focused on CpGs and genes reported to be associated with breast cancer risk in previous studies. Of the 250 CpGs reported by Xu et al. [26], 205 were included in our meta-analysis; we found no evidence of an association between these CpGs and breast cancer risk (*q* value > 0.17; *q* value calculation based on those 205 CpGs). Overall, methylation at the promoter region of *BRCA1* was not associated with breast cancer risk (*P* = 0.88); of the 26 CpGs in this region that we examined, pooled ORs were consistent in direction with previous reports for 14, with cg20185525 having the largest OR (OR = 1.11, 95% CI = 0.99, 1.25). With respect to gene *RASSF1A*, 43 CpGs were in its promoter region but only 7 of them were associated with breast cancer risk in the direction previously reported. Overall, there was no association with the promoter region of *RASSF1A* overall (enrichment *P* = 0.061). Of the 9 CpG sites located in the body of ATM, only two were associated with breast cancer risk in the direction previously reported and there was no enrichment (*P* = 0.26). Previously identified CpGs in *RPTOR* (cg06418238), *MGRN1* (cg00736299) and *RAPSN* (cg27466532) were not associated with breast cancer risk (cg06418238, *P* = 0.237, *I*^2^ = 0%; cg00736299, *P* = 0.779, *I*^2^ = 58%; cg27466532, *P* = 0.636, *I*^2^ = 0%).

### Associations between global methylation and breast cancer risk

**Fig. 2:**
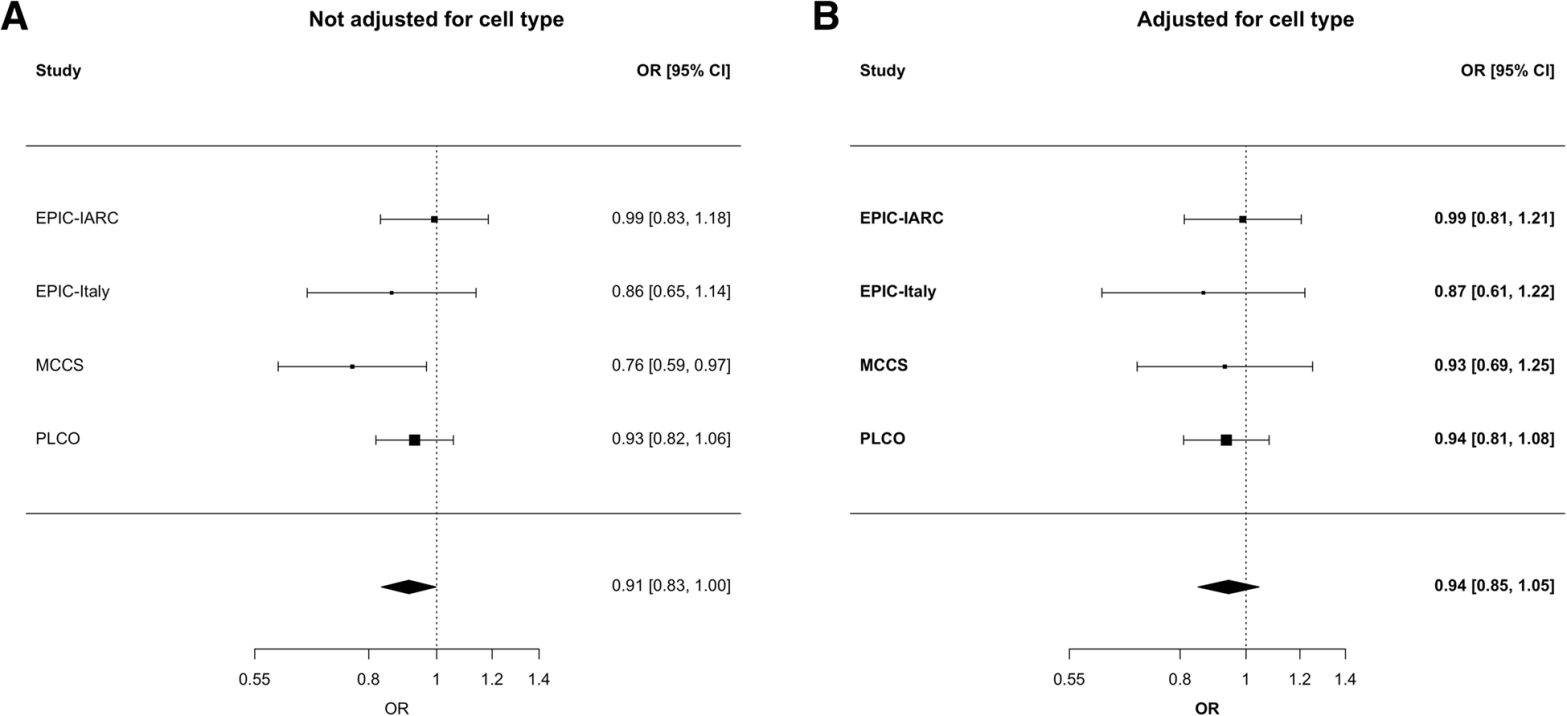
Average methylation and breast cancer risk. Forrest plot for the associations between average methylation and breast cancer risk when they are not adjusted (**a**) and adjusted (**b**) for cell type composition (CD8T+, CD4T+, NK, B cell, monocytes, granulocytes)

We found no evidence of an association between global methylation levels and breast cancer risk. We estimated an OR per one standard deviation increase in average methylation levels of 0.94 (95% CI 0.85, 1.05; *P* = 0.26; *P* for study heterogeneity = 0.86; *I*^2^ = 0%; Fig. 2). Neither adding previously published results from the NOWAC study (Additional file 1: Figure S1) nor stratifying by age at blood draw, ER status or time since blood draw qualitatively changed the result (Table 2). Global methylation was associated with breast cancer risk in women diagnosed with late-stage disease when analyses were not adjusted for cell type (OR = 0.83, 95% CI 0.71, 0.97), but not when cell types were included in the model (OR = 0.96, 95% CI 0.79, 1.13). Global methylation measures computed by the CpG region (island, shores, shelves, or open sea) were not associated with breast cancer risk. With respect to global methylation in CpG in promoters, a significant association was observed when the analysis was not adjusted for cell type, but it was not statistically significantly associated (*P* = 0.92) after adjustment (Table 2). Global methylation at other genomic regions, such as gene bodies, 3′UTR, or between genes, was not statistically significantly associated with breast cancer risk.

**Table 2:** Stratified analysis of average methylation levels and breast cancer risk by several characteristics

Characteristics	OR^*^	95% CI^*^	*P* value^*^	*P* hete	*I*^2^ (95% uncer. int.)	OR^§^	95% CI^§^	*P* value^§^	*P* hete	*I*^2^ (95% uncer. int.)
Age at blood draw (years)										
< 50	1.11	(0.82,1.50)	0.49	0.17	0.0 (0.0, 89.5)	1.04	(0.72, 1.51)	0.82	0.57	0.0 (−)
≥ 50	0.89	(0.81, 0.99)	0.03		0.0 (0.0, 75.8)	0.93	(0.84, 1.04)	0.22		0.0 (0.0, 0.0)
ER status										
ER+	0.92	(0.83, 1.02)	0.10	0.88	0.0 (0.0, 66.1)	0.93	(0.83, 1.05)	0.25	0.46	0.07 (0.0, 0.0)
ER−	0.93	(0.76, 1.15)	0.51		40.5 (0.0, 79.9)	1.04	(0.81, 1.34)	0.77		0.0 (0.0, 83.8)
Stage^†^										
Early	0.94	(0.84, 1.06)	0.34	0.20	0.0 (0.0, 76.0)	0.90	(0.79, 1.03)	0.13	0.68	0.0 (0.0, 72.4)
Late	0.83	(0.71, 0.97)	0.02		9.7 (0.0, 86.2)	0.95	(0.79, 1.13)	0.55		0.0 (0.0, 80.5)
Time since diagnosis										
< 2 years	0.96	(0.75, 1.24)	0.78	0.68	11.4 (0.0, 86.4)	0.99	(0.72, 1.37)	0.96	0.80	0.0 (0.0, 77.4)
≥ 2 years	0.91	(0.83, 1.00)	0.06		24.9 (0.0, 88.3)	0.95	(0.85, 1.06)	0.34		0.0 (0.0, 26.5)
Time since diagnosis										
< 5 years	0.93	(0.78, 1.10)	0.39	0.98	71.4 (18.6, 90.0)	0.93	(0.75, 1.14)	0.47	0.99	19.4 (0.0, 87.7)
≥ 5 and ≤ 10 years	0.92	(0.81, 1.04)	0.20		0.0 (0.0, 71.3)	0.94	(0.81, 1.08)	0.37		0.0 (0.0, 74.0)
> 10 years	0.94	(0.80, 1.11)	0.46		0.0 (0.0, 56.2)	0.93	(0.77, 1.12)	0.46		0.0 (0.0, 70.6)
CpG region^‡^										
CpG island	0.93	(0.85, 1.02)	0.13	0.98	0.0 (0.0, 79.6)	0.98	(0.87, 1.10)	0.74	0.95	0.0 (0.0, 71.1)
CpG shore	0.92	(0.84, 1.00)	0.06		0.0 (0.0, 23.0)	0.94	(0.84, 1.04)	0.23		0.0 (0.0, 0.0)
CpG shelf	0.94	(0.86, 1.03)	0.20		58.7 (0.0, 86.3)	0.96	(0.86, 1.06)	0.42		0.0 (0.0, 27.1)
Open sea	0.94	(0.86, 1.03)	0.18		62.3 (0.0, 87.3)	0.95	(0.85, 1.05)	0.31		0.0 (0.0, 60.9)
Regulatory region^¶^										
Promoter	0.90	(0.82, 0.99)	0.03	0.95	0.0 (0.0, 65.5)	0.93	(0.83, 1.04)	0.20	0.96	0.0 (0.0, 64.0)
Gene body	0.93	(0.85, 1.02)	0.14		48.1 (0.0, 82.8)	0.96	(0.87, 1.07)	0.46		0.0 (0.0, 62.3)
3′UTR	0.93	(0.85, 1.02)	0.12		59.8 (0.0, 86.6)	0.96	(0.87, 1.07)	0.50		0.0 (0.0, 50.8)
Intergenic	0.92	(0.84, 1.01)	0.07		53.7 (0.0, 84.7)	0.94	(0.85, 1.04)	0.27		0.0 (0.0, 64.4)

The *I*^2^ statistic estimates (in percent) how much of the total variability in the effect size estimates (which is composed of heterogeneity and sampling variability) can be attributed to heterogeneity among the true effects. *I*^2^ varies from 0 to 100%

*P* heterogeneity: Tests whether the variability in the observed effect sizes across strata is larger than would be expected based on sampling variability alone

^†^Stage: a cancer was considered an early stage if it was classified as localized (EPIC-Italy, EPIC-IARC) or stage I (MCCS, PLCO). A cancer was considered late-stage if it was classified as regional or metastatic (EPIC-Italy, EPIC-IARC) or stages II, III, or IV (MCCS, PLCO)

^‡^CpG region: shore 0–2 kb from CpG island, shelf = 2–4 kb from CpG island, OpenSea > 4 kb from CpG island

^¶^Based on the UCSC classification. CpGs in promoter: CpGs located in TSS200, TSS1500, 5′UTR, or exon 1 (TSS transcription start site)

^*^Adjusted for all variables except for cell type. ^§^Adjusted for all variables and cell type (CD8T+, CD4T+, NK, B cell, monocytes, granulocytes)

## Discussion

No association between DNA methylation in blood and breast cancer risk was observed in this meta-analysis, either at individual sites or globally. Associations with CpG sites that had previously been reported to be differentially methylated between breast cancer cases and controls by smaller studies were not replicated by our larger study.

*BRCA1* promoter methylation in blood has previously been reported to be higher in breast cancer patients compared with controls by some studies [1–3, 20], but not others [17, 18, 21]. In our study, we found no evidence of an association between BRCA1 methylation and breast cancer risk. It is important to note that blood collection in many of these previous studies was done at the time of cancer diagnosis, so DNA methylation could have been affected by the developing tumor; other studies included *BRCA1* mutation carriers that were oversampled/represented in the cases but not in the controls. This differs from this meta-analysis in which all studies were prospective and did not select on family history (less than 20% of participants reported having a family history of breast cancer in the PLCO, which was the only one of the four cohorts with available information). When analyses were restricted to subjects with a family history of breast cancer (108 cases and 107 controls), no significant association was observed. In addition, the assays used in the aforementioned studies were mostly based on targeted regions, for which a single measurement of methylation for an entire genomic region is obtained, and not genome-wide array-based assays, as in our study, for which methylation measurements are performed at individual CpG sites. However, we conducted enrichment tests and still did not observe associations. Similar results were observed for *RASSF1A*, despite previously reported associations [22, 42–44].

A few studies have reported hypermethylation of repetitive elements within the body of *ATM* to be associated with breast cancer risk [4, 15]. None of the CpG sites in *ATM* were statistically significantly associated with breast cancer risk in our study. However, the HM450 assay used in our analysis is gene-centered, with a special focus on gene promoters, and has less coverage of other areas of the genome, particularly repetitive elements. Our analysis included only nine CpG sites in the body of *ATM* so that we could not thoroughly assess gene-body methylation.

Previous findings for cg06418238 in *RPTOR*, cg00736299 in *MGRN1*, and cg27466532 in *RAPSN* that were recently reported to be associated with breast cancer risk [22] were not replicated by our study. One of the cohorts included in the meta-analysis (MCCS) had previously published the lack of replication of the CpG sites [24], and this was confirmed by this meta-analysis.

A previous analysis of the Sister Study reported a methylation signature for breast cancer consisting of 250 differentially methylated CpG sites in blood identified in a prospective study of 298 cases and 612 cancer-free women [26]. We found no evidence of association with risk for the 205 CpG sites we could examine in our larger study. A number of differences between the two studies could potentially explain the discrepant findings. First, the Sister Study was enriched for family history, with all cases and controls being relatives of breast cancer cases, whereas the four cohorts included in the meta-analysis were sampled from the general population. Another difference is that the average time from blood draw to diagnosis for cases in the Sister Study was 1.3 years, suggesting that some of these women may have been developing breast cancer at the time of blood draw. The average time to diagnosis for the cases in our study was over 5 years. None of the sites reported by the Sister Study were associated with risk. We also performed a sensitivity analysis to rule out the influence of potential subclinical disease.

There was no association between global DNA methylation and breast cancer risk in our meta-analysis. Previously, two of the cohorts (MCCS and EPIC-Italy) included in our study reported significant associations with breast cancer risk [23, 30]. However, EPIC-Italy included additional case-control pairs for the present analysis (248 pairs compared to 162 pairs in the previous publication [30]), and in this study, the association was no longer significant. Only one of the cohorts (MCCS) included in the meta-analysis showed a significant association, similar to their previously result, but only when the analysis was not adjusted for cell-type composition. No evidence of association was observed individually in either of the two largest, unpublished studies included in this meta-analysis (EPIC-IARC and PLCO), as was the case in the NOWAC study [30].

Previous studies that assessed associations between global DNA methylation and breast cancer risk have reached mixed conclusions. Global methylation (percentage of 5-methyldeoxycytosine, i.e., %5-dmC, with respect to the total cytosine content) was measured using different methods, at different genomic locations (LINE-1, CmCGG common in promoters, etc.), and various populations and study designs (retrospective versus prospective) were used, which makes it difficult to compare results. Several studies have also suggested that blood global hypomethylation is associated with increased cancer risk in general [9, 45–47], not necessarily just breast cancer risk. The reasons for any such association are not clear, but it may represent changes in the regulation of methyl transfer reactions due to age or certain exposures (residual confounding), the presence of the cancer years before diagnosis, circulating tumor DNA, or a false positive.

Our analysis has several strengths. First, it is the largest evaluation to date of blood DNA methylation and breast cancer risk. The large sample allowed us not only to investigate new associations, but also to test associations previously reported by smaller studies. Another important aspect of our analysis is the prospective design of all the studies included, which avoids the influence of reverse causation (methylation changes due to the presence of cancer), as well as potential effects on blood methylation of treatment given prior to blood collection. Our analysis also has some limitations. First, we had limited statistical power to detect small effects of methylation on breast cancer risk, despite having a sample size which was larger than that of previous studies which reported significant associations. Another limitation is that we restricted the analysis to sites in the Illumina HM450 BeadChip, which accounts for less than 2% of the 28 million CpG sites in the genome [48]. This array is also gene-centered and particularly lacks coverage in intergenic regions. Therefore, our analysis may have missed genomic regions that could be relevant to breast cancer risk. Finally, DNA methylation is cell-specific, and therefore, average methylation levels in blood cells could reflect cell heterogeneity by case-control status. None of the included studies had collected information on direct blood cell types, so we used a standard algorithm to infer blood cell counts and account for this source of heterogeneity.

## Conclusions

In summary, we found no evidence in this meta-analysis of four prospective cohort studies that DNA methylation measured in blood is associated with substantial variation in breast cancer risk. Larger studies may be required to determine if modest to weak associations exist between blood DNA methylation and breast cancer risk. Studies with greater methylation coverage of the genome may also have the potential to uncover novel associations.

## Additional file

Additional file 1: Description of the four prospective studies. Also, a figure with the results of the association between average methylation and breast cancer, adding published data from the NOWAC study. (DOCX 126 kb)
